# Association between deployment of a web-based electronic clinical decision support tool and antibiotic prescribing knowledge and confidence in an academic dental center

**DOI:** 10.1017/ash.2026.10825

**Published:** 2026-09-01

**Authors:** Michael J. Durkin, Danny Hanna, Susan Rowan, Tracey Habrock-Bach, Alice Bewley, Randi Foraker, Katie Suda

**Affiliations:** 1 Infectious Diseases, Washington University School of Medicine, St Louis, USA; 2 Division of Restorative Dentistry, University of Illinois Chicago College of Dentistry, USA; 3 University of Illinois Chicago College of Dentistry, USA; 4 Bioinformatics, Biostatistics, and Epidemiology, University of Missouri Columbia Health Care: MU Health Care, USA; 5 Center for Research on Healthcare, University of Pittsburgh School of Medicine, USA

## Abstract

**Objective:**

To evaluate antibiotic prescribing knowledge and confidence before versus after deployment of a web-based clinical decision support tool at an academic dental center.

**Design:**

Pre and post survey instrument.

**Setting:**

Academic dental center in the United States.

**Patients:**

Dental students, residents, and faculty.

**Methods:**

Participants completed a series of seven clinical vignettes assessing their antibiotic prescribing knowledge and confidence using 5-point Likert scale questions pre (2022) and post (2025) deployment of a web-based educational tool. The survey questions included content about antibiotic prophylaxis and treatment for dental infections. Mean Likert scale responses were compared before and after deployment of the tool. The responses were analyzed individually, pooled, and stratified by training level. Sensitivity analyses were conducted comparing results between tool users and non-users.

**Results:**

Two hundred and thirteen participants completed the pretool deployment survey, and 116 participants completed the post survey. Respondents were slightly less likely to prescribe antibiotics after having access to the tool (median pooled 5-point Likert Scale score 2.55 vs 2.67; *P* = .07). However, antibiotic prescribing confidence did not change significantly (median pooled score 3.37 vs 3.24; *P* = .21). Stratified results showed substantial variability based on individual vignette. Self-reported users of the tool (n = 37) demonstrated no differences in knowledge versus non-users (n = 97; *P* = .76) but did demonstrate a trend toward more confidence in their decisions (*P* = .07).

**Conclusions:**

Uptake of a web-based antibiotic prescribing tool was low overall and was not associated with any change in antibiotic prescribing knowledge and confidence. Additional interventions are needed to improve tool use in dental settings.

## Introduction

Dentists are responsible for 10% of outpatient antibiotic prescriptions in the United States (US),^1,2^ which is on par with the numbers dispensed by nurse practitioners and the rate prescribed by obstetrics and gynecology specialists. Antibiotic use in dentistry has been understudied, with a recent global systematic review identifying only three randomized controlled trials for antibiotic stewardship.^3^ The available US literature shows that about 64–83% of dental antibiotic prescriptions are likely unnecessary.^4,5,6^ Longitudinal trends show that antibiotic prescribing practices among dentists have remained unchanged over time, suggesting that there are substantial opportunities for improvement.^7,8,9^

Antibiotic stewardship in dentistry is important. Patients develop real harms with unnecessary antibiotic prescriptions from dental sources. Fifteen percent of outpatient *Clostridium difficile* infections (CDI) can be linked to a prior dental antibiotic prescription in the US.^10^ Additionally, a single dose of clindamycin for dental antibiotic prophylaxis contributes to fatal CDI each year,^11^ and all dental antibiotics have been associated with patient harms.^12^

Limited studies have evaluated how to improve antibiotic use in dental settings. Prior work has included projects in England and Australia, with audit and feedback reporting and clinical decision support (CDS).^13,14^ In the US, academic detailing has been successful at a small scale.^15,16^ Paper-based algorithms have also demonstrated improvements in antibiotic use as well.^17^ To our knowledge, no electronic clinical decision support systems have been tested in dental settings in the US. The purpose of this manuscript was to evaluate antibiotic prescribing knowledge and confidence before versus after deployment of a web-based clinical decision support tool at an academic dental center.

## Methods

In our prior work, we used a technology implementation framework to conduct a pre implementation assessment of antibiotic prescribing knowledge and confidence about antibiotic prescribing decisions to aid in developing a clinical decision support tool. The survey, deployed in 2021, was disseminated to all dental students, residents, and faculty at the University of Illinois Chicago (UIC) College of Dentistry. Details of the methods with this initial survey can be found in the accompanying publication.^6,18^

Based on the implementation assessment survey findings and qualitative interviews, our study team built a web-based CDS tool to aid in antibiotic prescribing. The tool was built based on existing paper tools, educational curriculum, and established guidelines. Then the tool was pilot tested and refined with eight dentists following a think-aloud protocol until no further recommendations were made regarding changes. The tool, which can be accessed at dds.tips, was then deployed and password protected during the study period and restricted to those affiliated with the UIC College of Dentistry and outside pilot testers. One educational in-person grand rounds was hosted in September of 2023 to share the tool throughout the university as well as three weekly reminder emails on how to access and login to the tool. The website was also incorporated into the annual antibiotic stewardship educational curriculum at UIC and flyers advertising the tool were posted in clinical spaces. Due to low adoption of the electronic tool during the mid-point of the study, the study team also added domains for dental pain management in November 2024.

In this manuscript, we assessed the association between tool access and respondents’ knowledge and confidence in antibiotic prescribing by redeploying the survey instrument from our prior publication.^18^ Basic demographic information of study participants was collected. We compared survey respondents answers before vs after the survey using Wilcoxon ranked-sum testing. We analyzed both likelihood of and confidence in prescribing antibiotics based each clinical vignette. To ensure that the answers could be pooled, the optimal answer to each question was 1 (no antibiotics should be prescribed) and 5 (most confident). Answers were stratified by vignette and pooled into a total response rate over all seven vignettes. Sensitivity analyses were also completed stratifying answers based on training level and tool users vs non-users. Scatter plots were also performed to look at the correlation between antibiotic knowledge and confidence overall and stratified by training level. Pearson correlation coefficients were used to determine statistical significance.

## Results

We had 213 participants complete the knowledge and confidence surveys in the pre implementation period and 116 complete the survey in the post period. Information about the clinical training level of survey respondents is displayed in Table 1. Due to the release date of both the pre and post-CDS surveys, no Advanced-Standing dental students level 3s were included. In the post-release group, 37 (32%) reported that they had utilized the tool.

**Table 1.** Demographics of survey respondents Table 1 long description.

Training Level	Pre, N (%)	Post, N (%)
D1	41 (19%)	21 (18%)
D2	19 (9%)	18 (15%)
D3	30 (14%)	8 (7%)
D4	25 (12%)	10 (8%)
AS2	23 (11%)	15 (13%)
AS3	0 (0%)	0 (0%)
AS4	11 (5%)	11 (9%)
Resident	27 (13%)	14 (12%)
Faculty	35 (16%)	19 (16%)

Note. D1 = first year dental student, D2 = second year dental student, D3 = third year dental student, D4 = fourth year dental student; AS2 = advanced standing non-US dental student; AS3 = advanced standing non-US dental student; AS4 = advanced standing non-US dental student. Two students in the presurvey group did not document a training level. One resident in the postsurvey group did not indicate a specialty. Residents included the following specialties: Endodontics (pre N = 2; post N = 3), Pediatric Dentistry (N = 3; N = 0), Periodontics (N = 4; N = 3), Prosthodontics (N = 1; N = 1), Orthodontics (N = 11; N = 4), and Oral and Maxillofacial Surgery (N = 6; N = 2). Faculty included the following specialties: Endodontics (N = 2; N = 1), General Dentistry (N = 13; N = 0), Pediatric Dentistry (N = 4; N = 3), Periodontics (N = 6; N = 6), Oral Medicine (N = 2; N = 0), Oral and Maxillofacial Surgery (N = 1; N = 0), Orthodontics (N = 1; N = 1), Prosthodontics (N = 5; N = 0), Dental Public Health (N = 1; N = 0), Dental Hygienist (N = 0; N = 1), and Restorative Dentistry (N = 0; N = 6).

Comparisons of survey respondents’ knowledge pre and postdeployment of the tool are displayed in Table 2. Likelihood to prescribe antibiotics increased in 3 vignettes and decreased in four vignettes – with an overall non-significant trend toward less antibiotic prescribing (*P* = .07) postdeployment. Deployment of the tool was not associated with any increase in antibiotic prescribing confidence (*P* = .21).

**Table 2.** Likelihood of prescribing antibiotics and confidence in decision by scenario and overall Table 2 long description.

Vignette number	Mean ± SD Abx Rx			Mean ± SD Confidence		
	Pre (n=213)	Post (n=116)	*P* value	Pre (n=213)	Post (n=116)	*P* value
Vignette 1: prosthetic joint infection prophylaxis	2.69 ± 1.12	2.96 ± .94	.014	3.21 ± 1.17	3.33 ± .96	.582
Vignette 2: acute pericoronitis	3.1 ± 1.14	2.67 ± .97	<.001	3.32 ± 1.07	3.3 ± .96	.724
Vignette 3: acute oral pain	2.57 ± 1.1	2.07 ± .94	<.001	3.21 ± 1.16	3.42 ± 1.06	.149
Vignette 4: endocarditis prophylaxis	2.19 ± 1.01	2.7 ± 1.08	<.001	3.15 ± 1.15	3.4 ± .98	.049
Vignette 5: acute oral pain	2.08 ± 1.06	2.9 ± 1.2	<.001	3.4 ± 1.2	3.18 ± 1.12	.060
Vignette 6: prosthetic joint infection prophylaxis	3.17 ± 1.22	2.44 ± 1.05	<.001	3.26 ± 1.13	3.35 ± .96	.667
Vignette 7: endocarditis prophylaxis	2.86 ± 1.24	2.14 ± .99	<.001	3.09 ± 1.16	3.6 ± 1.05	<.001
Average likelihood across all seven vignettes	2.67 ± .55	2.55 ± .54	.071	3.24 ± .97	3.37 ± .83	.211

Note. Average likelihood is a mean of all scenarios and ranges from 1 to 5 (analogous to the original scale), where higher values indicate higher likelihood of prescribing antibiotics overall. Perfect score for vignettes is 1.00. *P* values generated from Mann–Whitney test.

Results in antibiotic prescribing knowledge and confidence stratified by training level are displayed in Table 3. Overall, likelihood of prescribing antibiotics was similar between dental students, residents, and faculty (*P* = .08). However, confidence in antibiotic prescribing decisions was higher in a stepwise fashion with students being the least confident, followed by residents, and faculty as the most confident (*P* < .01).

**Table 3.**
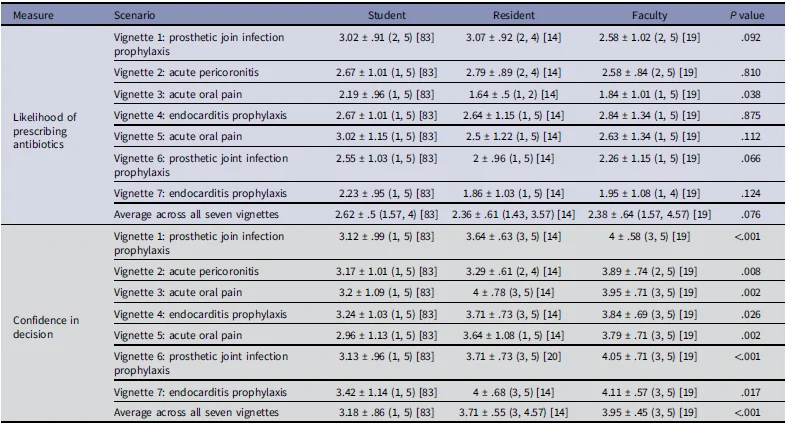
Comparison of postclinical decision support tool average likelihood of antibiotic prescribing and confidence in decision across students, residents, and faculty for each scenario and overall Table 3 long description.

Note. Statistics reported as mean ± standard deviation (range) [n]; *P* values generated from Kruskal-Wallis test for individual scenarios and ANOVA for average across all scenarios.

Detailed Likert Scale responses based on confidence and knowledge in antibiotic prescribing are provided in supplement tables 1 and 2, respectively. Supplement Table 3 displays stratified results based on training level. The results are further stratified for dental students into clinical and preclinical years in Supplement Table 4. Supplement Table 5 displays the comparison of confidence in decision among students, residents, and faculty for each scenario and overall. Supplement Table 6 provides the same results but with dental students stratified as clinical and preclinical. Supplement Table 7 and 8 compares respondents’ knowledge and confidence in antibiotic prescribing between CDSS self-reported users and non-users. This showed that user knowledge and confidence was no different from non-users overall and by scenario.

Among all participants, we observed that individuals who were less confident were more likely to prescribe antibiotics (*P* = .03; Supplement Figure 1). However, this correlation was not observed in our results when stratified by training level (Supplement Figures 2–5).

## Discussion

To our knowledge, this is the first study that evaluated a web-based CDS tool on antibiotic prescribing knowledge and confidence in dental settings in the US, which is now openly available at dds.tips. Our findings demonstrate that releasing a web-based tool was not associated with an increase in confidence or knowledge in antibiotic prescribing among survey respondents, despite regular reminders and education at an academic dental center.

Our findings reinforced the need for dental antibiotic stewardship. The survey responses show that respondents are not particularly confident about their antibiotic prescribing decisions and frequently continue to prescribe antibiotics when they are not necessary based on clinical vignettes. Furthermore, our pooled and correlation results showed that less confident dentists are more likely to prescribe antibiotics—further suggesting that additional education or CDS tools would be beneficial.

However, our findings suggest that additional work, beyond providing a web-based tool, may be necessary to improve antibiotic prescribing confidence and practices. Prior studies have demonstrated that academic detailing and audit and feedback approaches may be beneficial in dentistry.^13,19,20^ However, simple interventions such as paper-based tools also appear to be effective at reducing antibiotic prescribing in dental settings.^17^ It is likely that some of these interventions will be more effective when combined or modified using human-centered design approaches. For example, dentists often feel pressure to prescribe antibiotics when they are recommended by physicians, even when they know it is wrong.^21^ Many antibiotics prescribed in dental settings may fall outside of guidelines, which would fall outside of our tools.^21^ Several additional factors, such as patient expectations, fear, treatment delays, and diagnostic uncertainty also play a role in antibiotic prescribing according to a recent meta analysis.^22^

There may have been unique limitations with this study that limited the strength of our findings. We deployed the tool in the first US dental school to implement the Centers for Disease Control and Prevention Core Elements of Outpatient Antibiotic Stewardship. This may have limited the opportunities for additional improvements in antibiotic use. Anecdotally, some of our earlier qualitative work when deploying the tool included quotes from students that they infrequently or have never prescribed antibiotics.^19^ It is also possible that the login requirements reduced utilization from study participants. Low utilization could for a variety of reasons. First, the tool may not have been user friendly. We think that this is unlikely as pilot testing was performed and some aspects of the tool were based on existing online and paper resources. Second, the tool may have been cumbersome from a workflow perspective. This is very possible, as our prior qualitative work suggested that some students preferred a tool that was embedded into the electronic health record.^19^ Third, low uptake could have been a sign of little need for the tool. As discussed before, prior interviews also highlighted that several students reported rarely or never prescribing antibiotics in their training. This lack of prior antibiotic exposure may have decreased the likelihood that dentists would consider opening the tool to help with antibiotic prescribing. Fourth, the tool was deployed alongside a longstanding paper tool available chairside, which may have been easier for participants to access. Fifth, some of the low utilization may be because students have a faculty member present overseeing their work. In this scenario, it may be easier for dental students and residents to simply ask their attending, rather than find their phone or a computer to access the tool. Sixth, it is also possible that the tool needed to be embedded into the electronic health record system, which is what most participants desired during our interviews. Seventh, uptake of the tool was far lower than anticipated and did not improve substantially during the study period. Our study team added new features, including a pain module, without much uptake in users. Eight, here were temporal differences in the study time line, with earlier participants completing the survey 3 years before people completing it in the post period. This, in theory, could contribute to artificially increasing the likelihood of detecting a positive finding based on continued general education about antibiotic stewardship in dentistry. However, our null findings imply that this is unlikely. Ninth, we had originally anticipated tracking users over time using a combination of university and personal email addresses with web registration to measure implementation outcomes. Due to both partial and duplicate registrations, tracking users by website metrics was challenging. Finally, our study was also performed at a single academic dental center, and the results may have been different in diverse settings. Additional work, probably combining academic detailing with CDS tools, integrating CDS into the electronic medical record, or providing just-in-time education based on the clinical scenario should be explored.

In conclusion, releasing a web-based CDS tool at an academic dental center was not associated with an increase in dental provider knowledge and confidence in antibiotic prescribing decisions. Additional research identifying modified, additional, or combined interventions are needed to improve antibiotic prescribing in dental settings.

## Supporting information

10.1017/ash.2026.10825.sm001 Durkin et al. supplementary material Durkin et al. supplementary material

## Supplementary material

The supplementary material for this article is available at https://doi.org/10.1017/ash.2026.10825.

## Table 1. Long description

A table titled ʼDemographics of survey respondentsʼ displays the distribution of survey respondents by training level before and after implementation. The table has 9 rows and 3 columns. The columns are labeled ʼTraining Levelʼ, ʼPre, N (%)ʼ, and ʼPost, N (%)ʼ. The rows are labeled with different training levels: D1, D2, D3, D4, AS2, AS3, AS4, Resident, and Faculty. Each row shows the number and percentage of respondents at each training level before and after implementation. Row 1: D1, Pre: 41 (19 percent), Post: 21 (18 percent). Row 2: D2, Pre: 19 (9 percent), Post: 18 (15 percent). Row 3: D3, Pre: 30 (14 percent), Post: 8 (7 percent). Row 4: D4, Pre: 25 (12 percent), Post: 10 (8 percent). Row 5: AS2, Pre: 23 (11 percent), Post: 15 (13 percent). Row 6: AS3, Pre: 0 (0 percent), Post: 0 (0 percent). Row 7: AS4, Pre: 11 (5 percent), Post: 11 (9 percent). Row 8: Resident, Pre: 27 (13 percent), Post: 14 (12 percent). Row 9: Faculty, Pre: 35 (16 percent), Post: 19 (16 percent). The table shows the number and percentage of respondents at each training level before and after implementation, highlighting changes in the distribution of respondents across different training levels.

Navigate back to Table 1..

## Table 2. Long description

A table with seven rows and four columns comparing the likelihood of prescribing antibiotics and confidence in the decision across different scenarios before and after deployment of a tool. The columns are labeled ʼVignette numberʼ, ʼMean ± SD Abx Rxʼ, and ʼMean ± SD Confidenceʼ. The ʼMean ± SD Abx Rxʼ column is further divided into ʼPre (n=213)ʼ, ʼPost (n=116)ʼ, and ʼP valueʼ. The ʼMean ± SD Confidenceʼ column is divided into ʼPre (n=213)ʼ, ʼPost (n=116)ʼ, and ʼP valueʼ. Each row corresponds to a different vignette scenario. Row 1: Vignette 1: prosthetic joint infection prophylaxis, Pre 2.69 ± 1.12, Post 2.96 ± .94, P value .014, Pre 3.21 +/- 1.17, Post 3.33 ± .96, P value .582. Row 2: Vignette 2: acute pericoronitis, Pre 3.1 ± 1.14, Post 2.67 ± .97, P value <.001, Pre 3.32 ± 1.07, Post 3.3 ± .96, P value .724. Row 3: Vignette 3: acute oral pain, Pre 2.57 ± 1.1, Post 2.07 ± .94, P value <.001, Pre 3.21 ± 1.16, Post 3.42 ± 1.06, P value .149. Row 4: Vignette 4: endocarditis prophylaxis, Pre 2.19 ± 1.01, Post 2.7 ± 1.08, P value <.001, Pre 3.15 ± 1.15, Post 3.4 ± .98, P value .049. Row 5: Vignette 5: acute oral pain, Pre 2.08 ± 1.06, Post 2.9 ± 1.2, P value <.001, Pre 3.4 ± 1.2, Post 3.18 ± 1.12, P value .060. Row 6: Vignette 6: prosthetic joint infection prophylaxis, Pre 3.17 ± 1.22, Post 2.44 ± 1.05, P value <.001, Pre 3.26 ± 1.13, Post 3.35 ± .96, P value .667. Row 7: Vignette 7: endocarditis prophylaxis, Pre 2.86 ± 1.24, Post 2.14 ± .99, P value <.001, Pre 3.09 ± 1.16, Post 3.6 ± 1.05, P value <.001. The last row shows the average likelihood across all seven vignettes, Pre 2.67 ± .55, Post 2.55 ± .54, P value .071, Pre 3.24 ± .97, Post 3.37 ± .83, P value .211.

Navigate back to Table 2..

## Table 3. Long description

A table comparing the likelihood of prescribing antibiotics and confidence in decision across students, residents, and faculty for different scenarios. The table has 14 rows and 6 columns. The columns are labeled Measure, Scenario, Student, Resident, Faculty, and P value. The rows are grouped under two main measures: Likelihood of prescribing antibiotics and Confidence in decision. Each measure includes seven scenarios and an average across all seven vignettes. The scenarios include prosthetic joint infection prophylaxis, acute pericoronitis, acute oral pain, endocarditis prophylaxis, and more. The values represent the mean and standard deviation of the likelihood or confidence scores, with the number of participants in parentheses. The P value indicates the statistical significance of the differences between the groups. Notable trends include higher confidence in decision among faculty compared to residents and students, with P values indicating significant differences in some scenarios.

Navigate back to Table 3..
