# Supplementary material for: Association between deployment of a web-based electronic clinical decision support tool and antibiotic prescribing knowledge and confidence in an academic dental center
Source: Antimicrob Steward Healthc Epidemiol. 2026 Sep 1;6(1):e247. doi: 10.1017/ash.2026.10825 (PMC13760301; doi:10.1017/ash.2026.10825)
Supplement: Supplementary file 1 [file S2732494X26108250sup001.docx]

**Supplemental Materials.**

**Supplement Table 1. Descriptive statistics for likelihood of prescribing antibiotics by scenario and overall**

| **Label** | **Never** | **Unlikely** | **Maybe** | **Probably** | **Always** | **Mean +/- SD** |
| --- | --- | --- | --- | --- | --- | --- |
| Scenario 1: Likelihood of prescribing antibiotics | 0 (0%) | 49 (42.2%) | 27 (23.3%) | 36 (31%) | 4 (3.5%) | 2.96 +/- 0.94 |
| Scenario 2: Likelihood of prescribing antibiotics | 7 (6%) | 54 (46.6%) | 29 (25%) | 22 (19%) | 4 (3.5%) | 2.67 +/- 0.97 |
| Scenario 3: Likelihood of prescribing antibiotics | 30 (25.9%) | 61 (52.6%) | 16 (13.8%) | 5 (4.3%) | 4 (3.5%) | 2.07 +/- 0.94 |
| Scenario 4: Likelihood of prescribing antibiotics | 11 (9.5%) | 50 (43.1%) | 25 (21.6%) | 23 (19.8%) | 7 (6%) | 2.7 +/- 1.08 |
| Scenario 5: Likelihood of prescribing antibiotics | 9 (7.8%) | 47 (40.5%) | 22 (19%) | 23 (19.8%) | 15 (12.9%) | 2.9 +/- 1.2 |
| Scenario 6: Likelihood of prescribing antibiotics | 17 (14.7%) | 58 (50%) | 19 (16.4%) | 17 (14.7%) | 5 (4.3%) | 2.44 +/- 1.05 |
| Scenario 7: Likelihood of prescribing antibiotics | 31 (26.7%) | 54 (46.6%) | 17 (14.7%) | 12 (10.3%) | 2 (1.7%) | 2.14 +/- 0.99 |
| Average likelihood of prescribing antibiotics across all 7 scenarios | | | | | | 2.55 +/- 0.54 |

Average likelihood is a mean of all scenarios and ranges from 1-5 (analogous to the original scale), where higher values indicate higher likelihood of prescribing antibiotics overall

**Supplement Table 2. Descriptive statistics for confidence in antibiotic prescribing decision by scenario and overall**

| **Label** | **Not confident at all** | **Slightly confident** | **Moderately confident** | **Very confident** | **Extremely confident** | **Mean +/- SD** |
| --- | --- | --- | --- | --- | --- | --- |
| Scenario 1: Confidence in decision | 5 (4.3%) | 15 (12.9%) | 43 (37.1%) | 43 (37.1%) | 10 (8.6%) | 3.33 +/- 0.96 |
| Scenario 2: Confidence in decision | 4 (3.5%) | 19 (16.4%) | 41 (35.3%) | 42 (36.2%) | 10 (8.6%) | 3.3 +/- 0.96 |
| Scenario 3: Confidence in decision | 7 (6%) | 12 (10.3%) | 39 (33.6%) | 41 (35.3%) | 17 (14.7%) | 3.42 +/- 1.06 |
| Scenario 4: Confidence in decision | 4 (3.5%) | 15 (12.9%) | 42 (36.2%) | 41 (35.3%) | 14 (12.1%) | 3.4 +/- 0.98 |
| Scenario 5: Confidence in decision | 11 (9.5%) | 16 (13.8%) | 44 (37.9%) | 31 (26.7%) | 14 (12.1%) | 3.18 +/- 1.12 |
| Scenario 6: Confidence in decision | 4 (3.5%) | 16 (13.8%) | 43 (37.1%) | 41 (35.3%) | 12 (10.3%) | 3.35 +/- 0.96 |
| Scenario 7: Confidence in decision | 6 (5.2%) | 11 (9.5%) | 27 (23.3%) | 51 (44%) | 21 (18.1%) | 3.6 +/- 1.05 |
| Average confidence in decision across all 7 scenarios | | | | | | 3.37 +/- 0.83 |

Average confidence is a mean of all scenarios and ranges from 1-5 (analogous to the original scale), where higher values indicate higher confidence overall

**Supplement Table 3. Comparison of likelihood of antibiotic prescribing among students, residents, and faculty for each scenario and overall**

| **Label** | **Group** | **Student** | **Resident** | **Faculty** | **p-value** |
| --- | --- | --- | --- | --- | --- |
| Scenario 1: Likelihood of prescribing antibiotics | Never | 0/83 (0%) | 0/14 (0%) | 13/19 (68.4%) | 0.034 |
|  | Unlikely | 31/83 (37.3%) | 5/14 (35.7%) | 3/19 (15.8%) |  |
|  | Maybe | 21/83 (25.3%) | 3/14 (21.4%) | 1/19 (5.3%) |  |
|  | Probably | 29/83 (34.9%) | 6/14 (42.9%) | 2/19 (10.5%) |  |
|  | Always | 2/83 (2.4%) | 0/14 (0%) | 0/19 (0%) |  |
| Scenario 2: Likelihood of prescribing antibiotics | Never | 7/83 (8.4%) | 0/14 (0%) | 0/19 (0%) | 0.565 |
|  | Unlikely | 36/83 (43.4%) | 7/14 (50%) | 11/19 (57.9%) |  |
|  | Maybe | 20/83 (24.1%) | 3/14 (21.4%) | 6/19 (31.6%) |  |
|  | Probably | 17/83 (20.5%) | 4/14 (28.6%) | 1/19 (5.3%) |  |
|  | Always | 3/83 (3.6%) | 0/14 (0%) | 1/19 (5.3%) |  |
| Scenario 3: Likelihood of prescribing antibiotics | Never | 17/83 (20.5%) | 5/14 (35.7%) | 8/19 (42.1%) | 0.344 |
|  | Unlikely | 44/83 (53%) | 9/14 (64.3%) | 8/19 (42.1%) |  |
|  | Maybe | 14/83 (16.9%) | 0/14 (0%) | 2/19 (10.5%) |  |
|  | Probably | 5/83 (6%) | 0/14 (0%) | 0/19 (0%) |  |
|  | Always | 3/83 (3.6%) | 0/14 (0%) | 1/19 (5.3%) |  |
| Scenario 4: Likelihood of prescribing antibiotics | Never | 7/83 (8.4%) | 1/14 (7.1%) | 3/19 (15.8%) | 0.394 |
|  | Unlikely | 36/83 (43.4%) | 8/14 (57.1%) | 6/19 (31.6%) |  |
|  | Maybe | 20/83 (24.1%) | 1/14 (7.1%) | 4/19 (21.1%) |  |
|  | Probably | 17/83 (20.5%) | 3/14 (21.4%) | 3/19 (15.8%) |  |
|  | Always | 3/83 (3.6%) | 1/14 (7.1%) | 3/19 (15.8%) |  |
| Scenario 5: Likelihood of prescribing antibiotics | Never | 4/83 (4.8%) | 2/14 (14.3%) | 3/19 (15.8%) | 0.143 |
|  | Unlikely | 30/83 (36.1%) | 8/14 (57.1%) | 9/19 (47.4%) |  |
|  | Maybe | 20/83 (24.1%) | 0/14 (0%) | 2/19 (10.5%) |  |
|  | Probably | 18/83 (21.7%) | 3/14 (21.4%) | 2/19 (10.5%) |  |
|  | Always | 11/83 (13.3%) | 1/14 (7.1%) | 3/19 (15.8%) |  |
| Scenario 6: Likelihood of prescribing antibiotics | Never | 9/83 (10.8%) | 3/14 (21.4%) | 5/19 (26.3%) | 0.137 |
|  | Unlikely | 40/83 (48.2%) | 10/14 (71.4%) | 8/19 (42.1%) |  |
|  | Maybe | 16/83 (19.3%) | 0/14 (0%) | 3/19 (15.8%) |  |
|  | Probably | 15/83 (18.1%) | 0/14 (0%) | 2/19 (10.5%) |  |
|  | Always | 3/83 (3.6%) | 1/14 (7.1%) | 1/19 (5.3%) |  |
| Scenario 7: Likelihood of prescribing antibiotics | Never | 18/83 (21.7%) | 5/14 (35.7%) | 8/19 (42.1%) | 0.109 |
|  | Unlikely | 39/83 (47%) | 8/14 (57.1%) | 7/19 (36.8%) |  |
|  | Maybe | 16/83 (19.3%) | 0/14 (0%) | 1/19 (5.3%) |  |
|  | Probably | 9/83 (10.8%) | 0/14 (0%) | 3/19 (15.8%) |  |
|  | Always | 1/83 (1.2%) | 1/14 (7.1%) | 0/19 (0%) |  |
| Average likelihood of prescribing antibiotics across all 7 scenarios |  | 2.62 +/- 0.5 (1.57, 4) [83] | 2.36 +/- 0.61 (1.43, 3.57) [14] | 2.38 +/- 0.64 (1.57, 4.57) [19] | 0.076 |

For categorical variables, statistics reported as Frequency/Group Total (Column %) and p-values generated using Chi-Square or Fisher's Exact tests. For continuous variable, statistics reported as mean +/- standard deviation (range) [n] and p-values generated using ANOVA.

**Supplement Table 4. Comparison of likelihood of antibiotic prescribing among pre-clinical students, clinical students, and residents for each scenario and overall**

| **Label** | **Group** | **Student: D1, D2, AS2** | **Student: D3, D4, AS4** | **Resident (all specialties)** | **p-value** |
| --- | --- | --- | --- | --- | --- |
| Scenario 1: Likelihood of prescribing antibiotics | Never | 0/54 (0%) | 0/29 (0%) | 0/14 (0%) | 0.655 |
|  | Unlikely | 21/54 (38.9%) | 10/29 (34.5%) | 5/14 (35.7%) |  |
|  | Maybe | 13/54 (24.1%) | 8/29 (27.6%) | 3/14 (21.4%) |  |
|  | Probably | 20/54 (37%) | 9/29 (31%) | 6/14 (42.9%) |  |
|  | Always | 0/54 (0%) | 2/29 (6.9%) | 0/14 (0%) |  |
| Scenario 2: Likelihood of prescribing antibiotics | Never | 3/54 (5.6%) | 4/29 (13.8%) | 0/14 (0%) | 0.287 |
|  | Unlikely | 26/54 (48.1%) | 10/29 (34.5%) | 7/14 (50%) |  |
|  | Maybe | 14/54 (25.9%) | 6/29 (20.7%) | 3/14 (21.4%) |  |
|  | Probably | 11/54 (20.4%) | 6/29 (20.7%) | 4/14 (28.6%) |  |
|  | Always | 0/54 (0%) | 3/29 (10.3%) | 0/14 (0%) |  |
| Scenario 3: Likelihood of prescribing antibiotics | Never | 11/54 (20.4%) | 6/29 (20.7%) | 5/14 (35.7%) | 0.580 |
|  | Unlikely | 30/54 (55.6%) | 14/29 (48.3%) | 9/14 (64.3%) |  |
|  | Maybe | 9/54 (16.7%) | 5/29 (17.2%) | 0/14 (0%) |  |
|  | Probably | 3/54 (5.6%) | 2/29 (6.9%) | 0/14 (0%) |  |
|  | Always | 1/54 (1.9%) | 2/29 (6.9%) | 0/14 (0%) |  |
| Scenario 4: Likelihood of prescribing antibiotics | Never | 5/54 (9.3%) | 2/29 (6.9%) | 1/14 (7.1%) | 0.261 |
|  | Unlikely | 22/54 (40.7%) | 14/29 (48.3%) | 8/14 (57.1%) |  |
|  | Maybe | 17/54 (31.5%) | 3/29 (10.3%) | 1/14 (7.1%) |  |
|  | Probably | 9/54 (16.7%) | 8/29 (27.6%) | 3/14 (21.4%) |  |
|  | Always | 1/54 (1.9%) | 2/29 (6.9%) | 1/14 (7.1%) |  |
| Scenario 5: Likelihood of prescribing antibiotics | Never | 2/54 (3.7%) | 2/29 (6.9%) | 2/14 (14.3%) | 0.273 |
|  | Unlikely | 17/54 (31.5%) | 13/29 (44.8%) | 8/14 (57.1%) |  |
|  | Maybe | 14/54 (25.9%) | 6/29 (20.7%) | 0/14 (0%) |  |
|  | Probably | 13/54 (24.1%) | 5/29 (17.2%) | 3/14 (21.4%) |  |
|  | Always | 8/54 (14.8%) | 3/29 (10.3%) | 1/14 (7.1%) |  |
| Scenario 6: Likelihood of prescribing antibiotics | Never | 4/54 (7.4%) | 5/29 (17.2%) | 3/14 (21.4%) | 0.016 |
|  | Unlikely | 25/54 (46.3%) | 15/29 (51.7%) | 10/14 (71.4%) |  |
|  | Maybe | 9/54 (16.7%) | 7/29 (24.1%) | 0/14 (0%) |  |
|  | Probably | 14/54 (25.9%) | 1/29 (3.4%) | 0/14 (0%) |  |
|  | Always | 2/54 (3.7%) | 1/29 (3.4%) | 1/14 (7.1%) |  |
| Scenario 7: Likelihood of prescribing antibiotics | Never | 8/54 (14.8%) | 10/29 (34.5%) | 5/14 (35.7%) | 0.015 |
|  | Unlikely | 24/54 (44.4%) | 15/29 (51.7%) | 8/14 (57.1%) |  |
|  | Maybe | 12/54 (22.2%) | 4/29 (13.8%) | 0/14 (0%) |  |
|  | Probably | 9/54 (16.7%) | 0/29 (0%) | 0/14 (0%) |  |
|  | Always | 1/54 (1.9%) | 0/29 (0%) | 1/14 (7.1%) |  |
| Average likelihood of prescribing antibiotics across all 7 scenarios |  | 2.67 +/- 0.48 (1.71, 4) [54] | 2.55 +/- 0.52 (1.57, 3.57) [29] | 2.36 +/- 0.61 (1.43, 3.57) [14] | 0.123 |

For categorical variables, statistics reported as Frequency/Group Total (Column %) and p-values generated using Chi-Square or Fisher's Exact tests. For continuous variable, statistics reported as mean +/- standard deviation (range) [n] and p-values generated using ANOVA.

**Supplement Table 5. Comparison of confidence in decision among students, residents, and faculty for each scenario and overall**

| **Label** | **Group** | **Student** | **Resident** | **Faculty** | **p-value** |
| --- | --- | --- | --- | --- | --- |
| Scenario 1: Confidence in decision | Not confident at all | 5/83 (6%) | 0/14 (0%) | 0/19 (0%) | 0.009 |
|  | Slightly confident | 15/83 (18.1%) | 0/14 (0%) | 0/19 (0%) |  |
|  | Moderately confident | 34/83 (41%) | 6/14 (42.9%) | 3/19 (15.8%) |  |
|  | Very confident | 23/83 (27.7%) | 7/14 (50%) | 13/19 (68.4%) |  |
|  | Extremely confident | 6/83 (7.2%) | 1/14 (7.1%) | 3/19 (15.8%) |  |
| Scenario 2: Confidence in decision | Not confident at all | 4/83 (4.8%) | 0/14 (0%) | 0/19 (0%) | 0.066 |
|  | Slightly confident | 17/83 (20.5%) | 1/14 (7.1%) | 1/19 (5.3%) |  |
|  | Moderately confident | 30/83 (36.1%) | 8/14 (57.1%) | 3/19 (15.8%) |  |
|  | Very confident | 25/83 (30.1%) | 5/14 (35.7%) | 12/19 (63.2%) |  |
|  | Extremely confident | 7/83 (8.4%) | 0/14 (0%) | 3/19 (15.8%) |  |
| Scenario 3: Confidence in decision | Not confident at all | 7/83 (8.4%) | 0/14 (0%) | 0/19 (0%) | 0.111 |
|  | Slightly confident | 12/83 (14.5%) | 0/14 (0%) | 0/19 (0%) |  |
|  | Moderately confident | 30/83 (36.1%) | 4/14 (28.6%) | 5/19 (26.3%) |  |
|  | Very confident | 25/83 (30.1%) | 6/14 (42.9%) | 10/19 (52.6%) |  |
|  | Extremely confident | 9/83 (10.8%) | 4/14 (28.6%) | 4/19 (21.1%) |  |
| Scenario 4: Confidence in decision | Not confident at all | 4/83 (4.8%) | 0/14 (0%) | 0/19 (0%) | 0.230 |
|  | Slightly confident | 15/83 (18.1%) | 0/14 (0%) | 0/19 (0%) |  |
|  | Moderately confident | 30/83 (36.1%) | 6/14 (42.9%) | 6/19 (31.6%) |  |
|  | Very confident | 25/83 (30.1%) | 6/14 (42.9%) | 10/19 (52.6%) |  |
|  | Extremely confident | 9/83 (10.8%) | 2/14 (14.3%) | 3/19 (15.8%) |  |
| Scenario 5: Confidence in decision | Not confident at all | 10/83 (12%) | 1/14 (7.1%) | 0/19 (0%) | 0.043 |
|  | Slightly confident | 16/83 (19.3%) | 0/14 (0%) | 0/19 (0%) |  |
|  | Moderately confident | 32/83 (38.6%) | 5/14 (35.7%) | 7/19 (36.8%) |  |
|  | Very confident | 17/83 (20.5%) | 5/14 (35.7%) | 9/19 (47.4%) |  |
|  | Extremely confident | 8/83 (9.6%) | 3/14 (21.4%) | 3/19 (15.8%) |  |
| Scenario 6: Confidence in decision | Not confident at all | 4/83 (4.8%) | 0/14 (0%) | 0/19 (0%) | 0.015 |
|  | Slightly confident | 16/83 (19.3%) | 0/14 (0%) | 0/19 (0%) |  |
|  | Moderately confident | 33/83 (39.8%) | 6/14 (42.9%) | 4/19 (21.1%) |  |
|  | Very confident | 25/83 (30.1%) | 6/14 (42.9%) | 10/19 (52.6%) |  |
|  | Extremely confident | 5/83 (6%) | 2/14 (14.3%) | 5/19 (26.3%) |  |
| Scenario 7: Confidence in decision | Not confident at all | 6/83 (7.2%) | 0/14 (0%) | 0/19 (0%) | 0.163 |
|  | Slightly confident | 11/83 (13.3%) | 0/14 (0%) | 0/19 (0%) |  |
|  | Moderately confident | 22/83 (26.5%) | 3/14 (21.4%) | 2/19 (10.5%) |  |
|  | Very confident | 30/83 (36.1%) | 8/14 (57.1%) | 13/19 (68.4%) |  |
|  | Extremely confident | 14/83 (16.9%) | 3/14 (21.4%) | 4/19 (21.1%) |  |
| Average confidence in decision across all 7 scenarios |  | 3.18 +/- 0.86 (1, 5) [83] | 3.71 +/- 0.55 (3, 4.57) [14] | 3.95 +/- 0.45 (3, 5) [19] | <0.001 |

For categorical variables, statistics reported as Frequency/Group Total (Column %) and p-values generated using Chi-Square or Fisher's Exact tests. For continuous variable, statistics reported as mean +/- standard deviation (range) [n] and p-values generated using ANOVA.

**Supplement Table 6. Comparison of confidence in decision among pre-clinical students, clinical students, and residents for each scenario and overall**

| **Label** | **Group** | **Student: D1, D2, AS2** | **Student: D3, D4, AS4** | **Resident (all specialties)** | **p-value** |
| --- | --- | --- | --- | --- | --- |
| Scenario 1: Confidence in decision | Not confident at all | 3/54 (5.6%) | 2/29 (6.9%) | 0/14 (0%) | 0.485 |
|  | Slightly confident | 12/54 (22.2%) | 3/29 (10.3%) | 0/14 (0%) |  |
|  | Moderately confident | 20/54 (37%) | 14/29 (48.3%) | 6/14 (42.9%) |  |
|  | Very confident | 15/54 (27.8%) | 8/29 (27.6%) | 7/14 (50%) |  |
|  | Extremely confident | 4/54 (7.4%) | 2/29 (6.9%) | 1/14 (7.1%) |  |
| Scenario 2: Confidence in decision | Not confident at all | 2/54 (3.7%) | 2/29 (6.9%) | 0/14 (0%) | 0.271 |
|  | Slightly confident | 13/54 (24.1%) | 4/29 (13.8%) | 1/14 (7.1%) |  |
|  | Moderately confident | 19/54 (35.2%) | 11/29 (37.9%) | 8/14 (57.1%) |  |
|  | Very confident | 18/54 (33.3%) | 7/29 (24.1%) | 5/14 (35.7%) |  |
|  | Extremely confident | 2/54 (3.7%) | 5/29 (17.2%) | 0/14 (0%) |  |
| Scenario 3: Confidence in decision | Not confident at all | 5/54 (9.3%) | 2/29 (6.9%) | 0/14 (0%) | 0.149 |
|  | Slightly confident | 10/54 (18.5%) | 2/29 (6.9%) | 0/14 (0%) |  |
|  | Moderately confident | 20/54 (37%) | 10/29 (34.5%) | 4/14 (28.6%) |  |
|  | Very confident | 16/54 (29.6%) | 9/29 (31%) | 6/14 (42.9%) |  |
|  | Extremely confident | 3/54 (5.6%) | 6/29 (20.7%) | 4/14 (28.6%) |  |
| Scenario 4: Confidence in decision | Not confident at all | 3/54 (5.6%) | 1/29 (3.4%) | 0/14 (0%) | 0.074 |
|  | Slightly confident | 14/54 (25.9%) | 1/29 (3.4%) | 0/14 (0%) |  |
|  | Moderately confident | 19/54 (35.2%) | 11/29 (37.9%) | 6/14 (42.9%) |  |
|  | Very confident | 12/54 (22.2%) | 13/29 (44.8%) | 6/14 (42.9%) |  |
|  | Extremely confident | 6/54 (11.1%) | 3/29 (10.3%) | 2/14 (14.3%) |  |
| Scenario 5: Confidence in decision | Not confident at all | 8/54 (14.8%) | 2/29 (6.9%) | 1/14 (7.1%) | 0.097 |
|  | Slightly confident | 13/54 (24.1%) | 3/29 (10.3%) | 0/14 (0%) |  |
|  | Moderately confident | 20/54 (37%) | 12/29 (41.4%) | 5/14 (35.7%) |  |
|  | Very confident | 7/54 (13%) | 10/29 (34.5%) | 5/14 (35.7%) |  |
|  | Extremely confident | 6/54 (11.1%) | 2/29 (6.9%) | 3/14 (21.4%) |  |
| Scenario 6: Confidence in decision | Not confident at all | 3/54 (5.6%) | 1/29 (3.4%) | 0/14 (0%) | 0.206 |
|  | Slightly confident | 12/54 (22.2%) | 4/29 (13.8%) | 0/14 (0%) |  |
|  | Moderately confident | 21/54 (38.9%) | 12/29 (41.4%) | 6/14 (42.9%) |  |
|  | Very confident | 17/54 (31.5%) | 8/29 (27.6%) | 6/14 (42.9%) |  |
|  | Extremely confident | 1/54 (1.9%) | 4/29 (13.8%) | 2/14 (14.3%) |  |
| Scenario 7: Confidence in decision | Not confident at all | 4/54 (7.4%) | 2/29 (6.9%) | 0/14 (0%) | 0.190 |
|  | Slightly confident | 9/54 (16.7%) | 2/29 (6.9%) | 0/14 (0%) |  |
|  | Moderately confident | 16/54 (29.6%) | 6/29 (20.7%) | 3/14 (21.4%) |  |
|  | Very confident | 20/54 (37%) | 10/29 (34.5%) | 8/14 (57.1%) |  |
|  | Extremely confident | 5/54 (9.3%) | 9/29 (31%) | 3/14 (21.4%) |  |
| Average confidence in decision across all 7 scenarios |  | 3.05 +/- 0.85 (1.14, 5) [54] | 3.41 +/- 0.86 (1, 5) [29] | 3.71 +/- 0.55 (3, 4.57) [14] | 0.015 |

For categorical variables, statistics reported as Frequency/Group Total (Column %) and p-values generated using Chi-Square or Fisher's Exact tests. For continuous variable, statistics reported as mean +/- standard deviation (range) [n] and p-values generated using ANOVA

**Supplement Table 7. Comparison of likelihood of antibiotic prescribing among clinical decision support tool users and non-users for each scenario and overall**

| **Label** | **Group** | **Non-Users** | **Users** | **p-value** |
| --- | --- | --- | --- | --- |
| Scenario 1: Likelihood of prescribing antibiotics | Never | 0 (0%) | 0 (0%) | 0.170 |
|  | Unlikely | 38/79 (48.1%) | 11/37 (29.7%) |  |
|  | Maybe | 15/79 (19%) | 12/37 (32.4%) |  |
|  | Probably | 24/79 (30.4%) | 12/37 (32.4%) |  |
|  | Always | 2/79 (2.5%) | 2/37 (5.4%) |  |
| Scenario 2: Likelihood of prescribing antibiotics | Never | 4/79 (5.1%) | 3/37 (8.1%) | 0.867 |
|  | Unlikely | 38/79 (48.1%) | 16/37 (43.2%) |  |
|  | Maybe | 18/79 (22.8%) | 11/37 (29.7%) |  |
|  | Probably | 16/79 (20.3%) | 6/37 (16.2%) |  |
|  | Always | 3/79 (3.8%) | 1/37 (2.7%) |  |
| Scenario 3: Likelihood of prescribing antibiotics | Never | 22/79 (27.8%) | 8/37 (21.6%) | 0.075 |
|  | Unlikely | 45/79 (57%) | 16/37 (43.2%) |  |
|  | Maybe | 9/79 (11.4%) | 7/37 (18.9%) |  |
|  | Probably | 1/79 (1.3%) | 4/37 (10.8%) |  |
|  | Always | 2/79 (2.5%) | 2/37 (5.4%) |  |
| Scenario 4: Likelihood of prescribing antibiotics | Never | 5/79 (6.3%) | 6/37 (16.2%) | 0.465 |
|  | Unlikely | 35/79 (44.3%) | 15/37 (40.5%) |  |
|  | Maybe | 18/79 (22.8%) | 7/37 (18.9%) |  |
|  | Probably | 15/79 (19%) | 8/37 (21.6%) |  |
|  | Always | 6/79 (7.6%) | 1/37 (2.7%) |  |
| Scenario 5: Likelihood of prescribing antibiotics | Never | 5/79 (6.3%) | 4/37 (10.8%) | 0.940 |
|  | Unlikely | 33/79 (41.8%) | 14/37 (37.8%) |  |
|  | Maybe | 15/79 (19%) | 7/37 (18.9%) |  |
|  | Probably | 16/79 (20.3%) | 7/37 (18.9%) |  |
|  | Always | 10/79 (12.7%) | 5/37 (13.5%) |  |
| Scenario 6: Likelihood of prescribing antibiotics | Never | 11/79 (13.9%) | 6/37 (16.2%) | 0.906 |
|  | Unlikely | 40/79 (50.6%) | 18/37 (48.6%) |  |
|  | Maybe | 12/79 (15.2%) | 7/37 (18.9%) |  |
|  | Probably | 13/79 (16.5%) | 4/37 (10.8%) |  |
|  | Always | 3/79 (3.8%) | 2/37 (5.4%) |  |
| Scenario 7: Likelihood of prescribing antibiotics | Never | 23/79 (29.1%) | 8/37 (21.6%) | 0.469 |
|  | Unlikely | 33/79 (41.8%) | 21/37 (56.8%) |  |
|  | Maybe | 12/79 (15.2%) | 5/37 (13.5%) |  |
|  | Probably | 10/79 (12.7%) | 2/37 (5.4%) |  |
|  | Always | 1/79 (1.3%) | 1/37 (2.7%) |  |
| Average likelihood of prescribing antibiotics across all 7 scenarios |  | 2.54 +/- 0.53 (1.71, 4.57) [79] | 2.58 +/- 0.58 (1.43, 4) [37] | 0.763 |

For categorical variables, statistics reported as Frequency/Group Total (Column %) and p-values generated using Chi-Square or Fisher's Exact tests. For continuous variable, statistics reported as mean +/- standard deviation (range) [n] and p-values generated using ANOVA.

**Supplement Table 8. Comparison of confidence in decision among clinical decision support tool users and non-users for each scenario and overall**

| **Label** | **Group** | **Non-Users** | **Users** | **p-value** |
| --- | --- | --- | --- | --- |
| Scenario 1: Confidence in decision | Not confident at all | 3/79 (3.8%) | 2/37 (5.4%) | 0.163 |
|  | Slightly confident | 14/79 (17.7%) | 1/37 (2.7%) |  |
|  | Moderately confident | 29/79 (36.7%) | 14/37 (37.8%) |  |
|  | Very confident | 26/79 (32.9%) | 17/37 (45.9%) |  |
|  | Extremely confident | 7/79 (8.9%) | 3/37 (8.1%) |  |
| Scenario 2: Confidence in decision | Not confident at all | 2/79 (2.5%) | 2/37 (5.4%) | 0.091 |
|  | Slightly confident | 17/79 (21.5%) | 2/37 (5.4%) |  |
|  | Moderately confident | 25/79 (31.6%) | 16/37 (43.2%) |  |
|  | Very confident | 30/79 (38%) | 12/37 (32.4%) |  |
|  | Extremely confident | 5/79 (6.3%) | 5/37 (13.5%) |  |
| Scenario 3: Confidence in decision | Not confident at all | 5/79 (6.3%) | 2/37 (5.4%) | 0.212 |
|  | Slightly confident | 11/79 (13.9%) | 1/37 (2.7%) |  |
|  | Moderately confident | 23/79 (29.1%) | 16/37 (43.2%) |  |
|  | Very confident | 30/79 (38%) | 11/37 (29.7%) |  |
|  | Extremely confident | 10/79 (12.7%) | 7/37 (18.9%) |  |
| Scenario 4: Confidence in decision | Not confident at all | 3/79 (3.8%) | 1/37 (2.7%) | 0.170 |
|  | Slightly confident | 14/79 (17.7%) | 1/37 (2.7%) |  |
|  | Moderately confident | 27/79 (34.2%) | 15/37 (40.5%) |  |
|  | Very confident | 25/79 (34.2%) | 16/37 (43.2%) |  |
|  | Extremely confident | 10/79 (12.7%) | 4/37 (10.8%) |  |
| Scenario 5: Confidence in decision | Not confident at all | 10/79 (12.7%) | 1/37 (2.7%) | 0.121 |
|  | Slightly confident | 13/79 (16.5%) | 3/37 (8.1%) |  |
|  | Moderately confident | 31/79 (39.2%) | 13/37 (35.1%) |  |
|  | Very confident | 17/79 (21.5%) | 14/37 (37.8%) |  |
|  | Extremely confident | 8/79 (10.1%) | 6/37 (16.2%) |  |
| Scenario 6: Confidence in decision | Not confident at all | 3/79 (3.8%) | 1/37 (2.7%) | 0.135 |
|  | Slightly confident | 15/79 (19%) | 1/37 (2.7%) |  |
|  | Moderately confident | 28/79 (35.4%) | 15/37 (40.5%) |  |
|  | Very confident | 26/79 (32.9%) | 15/37 (40.5%) |  |
|  | Extremely confident | 7/79 (8.9%) | 5/37 (13.5%) |  |
| Scenario 7: Confidence in decision | Not confident at all | 4/79 (5.1%) | 2/37 (5.4%) | 0.147 |
|  | Slightly confident | 11/79 (13.9%) | 0/37 (0%) |  |
|  | Moderately confident | 18/79 (22.8%) | 9/37 (24.3%) |  |
|  | Very confident | 33/79 (41.8%) | 18/37 (48.6%) |  |
|  | Extremely confident | 13/79 (16.5%) | 8/37 (21.6%) |  |
| Average confidence in decision across all 7 scenarios |  | 3.27 +/- 0.85 (1.14, 5) [79] | 3.57 +/- 0.76 (1, 5) [37] | 0.074 |

For categorical variables, statistics reported as Frequency/Group Total (Column %) and p-values generated using Chi-Square or Fisher's Exact tests. For continuous variable, statistics reported as mean +/- standard deviation (range) [n] and p-values generated using ANOVA

**Supplement Table 9. Comparison of average likelihood of antibiotic prescribing and confidence in decision across clinical decision support tool users and non-users for each scenario and overall**

| **Measure** | **Scenario** | **Non-Users** | **Users** | **p-value** |
| --- | --- | --- | --- | --- |
| Likelihood of prescribing antibiotics | Vignette 1: prosthetic join infection prophylaxis | 2.87 +/- 0.94 (2, 5) [79] | 3.14 +/- 0.92 (2, 5) [37] | 0.148 |
|  | Vignette 2: acute pericoronitis | 2.7 +/- 0.98 (1, 5) [79] | 2.62 +/- 0.95 (1, 5) [37] | 0.795 |
|  | Vignette 3: acute oral pain | 1.94 +/- 0.82 (1, 5) [79] | 2.35 +/- 1.11 (1, 5) [37] | 0.051 |
|  | Vignette 4: endocarditis prophylaxis | 2.77 +/- 1.07 (1, 5) [79] | 2.54 +/- 1.1 (1, 5) [37] | 0.300 |
|  | Vignette 5: acute oral pain | 2.91 +/- 1.18 (1, 5) [79] | 2.86 +/- 1.25 (1, 5) [37] | 0.829 |
|  | Vignette 6: prosthetic joint infection prophylaxis | 2.46 +/- 1.05 (1, 5) [79] | 2.41 +/- 1.07 (1, 5) [37] | 0.806 |
|  | Vignette 7: endocarditis prophylaxis | 2.15 +/- 1.03 (1, 5) [79] | 2.11 +/- 0.91 (1, 5) [37] | 0.972 |
|  | Average across all 7 vignettes | 2.54 +/- 0.53 (1.71, 4.57) [79] | 2.58 +/- 0.58 (1.43, 4) [37] | 0.763 |
| Confidence in decision | Vignette 1: prosthetic join infection prophylaxis | 3.25 +/- 0.98 (1, 5) [79] | 3.49 +/- 0.9 (1, 5) [37] | 0.165 |
|  | Vignette 2: acute pericoronitis | 3.24 +/- 0.95 (1, 5) [79] | 3.43 +/- 0.99 (1, 5) [37] | 0.331 |
|  | Vignette 3: acute oral pain | 3.37 +/- 1.08 (1, 5) [79] | 3.54 +/- 1.02 (1, 5) [37] | 0.511 |
|  | Vignette 4: endocarditis prophylaxis | 3.32 +/- 1.03 (1, 5) [79] | 3.57 +/- 0.83 (1, 5) [37] | 0.196 |
|  | Vignette 5: acute oral pain | 3 +/- 1.14 (1, 5) [79] | 3.57 +/- 0.96 (1, 5) [37] | 0.010 |
|  | Vignette 6: prosthetic joint infection prophylaxis | 3.24 +/- 0.99 (1, 5) [79] | 3.59 +/- 0.86 (1, 5) [37] | 0.068 |
|  | Vignette 7: endocarditis prophylaxis | 3.51 +/- 1.08 (1, 5) [79] | 3.81 +/- 0.97 (1, 5) [37] | 0.151 |
|  | Average across all 7 vignettes | 3.27 +/- 0.85 (1.14, 5) [79] | 3.57 +/- 0.76 (1, 5) [37] | 0.074 |

Statistics reported as mean +/- standard deviation (range) [n]; p-values generated from Kruskal-Wallis test for individual scenarios and ANOVA for average across all scenarios

**Supplement Figure 1. Correlation between likelihood of prescribing antibiotics and confidence in decision: Full sample**

**Supplement Figure 2. Correlation between likelihood of prescribing antibiotics and confidence in decision: Students only**

**Supplement Figure 3. Correlation between likelihood of prescribing antibiotics and confidence in decision: Residents only**

**Supplement Figure 4. Correlation between likelihood of prescribing antibiotics and confidence in decision: Faculty only**

**Supplement Figure 5. Correlation between likelihood of prescribing antibiotics and confidence in decision: Residents and Faculty only**
